# Некласические гормоны из семейства факторов роста фибробластов

**DOI:** 10.14341/probl13441

**Published:** 2024-11-04

**Authors:** С. А. Гронская, Н. В. Русяева, Ж. Е. Белая, Г. А. Мельниченко

**Affiliations:** Научный медицинский исследовательский центр эндокринологии; Научный медицинский исследовательский центр эндокринологии; Научный медицинский исследовательский центр эндокринологии; Научный медицинский исследовательский центр эндокринологии

**Keywords:** фактор роста фибробластов, ФРФ 23, ФРФ 19, ФРФ 21

## Abstract

Факторы роста фибробластов (ФРФ) — это группа сигнальных молекул, получивших свое название благодаря положительному влиянию на рост и размножение фибробластов. ФРФ оказывают свои эффекты преимущественно локально в тканях. Однако ФРФ 19, ФРФ 21 и ФРФ 23 секретируются в кровь и оказывают влияние на отдаленные органы и ткани, поэтому их можно классифицирвоать как неклассические гормоны. Биологическая роль ФРФ многообразна и зависит в большей степени от рецепторов и кофакторов, участвующих в передаче сигнала. ФРФ 19 и ФРФ 21 во­влечены в обмен глюкозы и липидов, а ФРФ 23 известен своим влиянием на обмен фосфора. ФРФ являются перспективными мишенями для разработки лекарственных средств. В настоящем обзоре суммируются современные знания о биологических эффектах неклассических гормонов из семейства ФРФ и потенциальные возможности их применения в качестве терапевтических мишеней.

## Abstract (en)

Fibroblast growth factors (FGFs) are a group of signaling molecules named for their ability to promote the growth and proliferation of fibroblasts and various other cell types. Typically, FGFs exert their effects locally by binding to receptors within the tissues where they are synthesized. However, certain members of this family, such as FGF 19, FGF 21, and FGF 23, diverge from this pattern. Following synthesis, these FGFs enter the bloodstream and act on distant organs and tissues by binding to their receptors and associated cofactors, thereby classified as non-classical hormones within the FGF family.

The biological functions of FGFs are diverse and contingent upon the specific receptors and cofactors involved in their signaling pathways. For instance, FGF 19 and FGF 21 play crucial roles in regulating glucose and lipid metabolism, whereas FGF 23 primarily influences phosphorus metabolism. Given their varied roles, FGFs present promising targets for therapeutic interventions and drug development.

This review aims to consolidate current understanding of FGF family hormones, elucidating their biological impacts and exploring their potential applications as therapeutic targets.

## Введение

Факторы роста фибробластов (ФРФ) (Fibroblast growth factors (FGF) — это белки, регулирующие множество биологических процессов, в частности, пролиферацию и дифференцировку клеток. ФРФ открыли в 1973 г., и был показан их стимулирующий эффект на рост фибробластов [1]. В состав семейства факторов роста фибробластов (ФРФ) входят 23 белка (рис. 1). Большинство ФРФ действуют паракринно, то есть оказывают влияние только на соседние клетки. Однако ФРФ 19, 21, 23 обладают эндокринными свойствами, секретируются в кровоток и воздействуют на специфичексие рецепторы в отдаленных органах и тканях [2]. ФРФ обладают структурным сходством, реагируют с рецепторами факторов роста фибробластов 1, 2, 3, 4 типов (ФРФР 1–4), (Fibroblast growth factor receptors 1, 2, 3, 4) (FGFR1–4)), участвуют в регуляции ангиогенеза, эмбриогенеза, регенерации [1]. ФРФ способны связываться с разными ФРФР. Специфичность и эффекты определяются многообразием комбинаций ФРФ + ФРФР + кофактор, которые в комплексе активируют специфические сигнальные пути. Например, для паракринных ФРФ в качестве кофактора выступает гепарин / гепаринсульфат, а для эндокринных ФРФ — белок Клото (klotho). Без наличия кофактора, аффинность ФРФ к рецепторам низкая.

ФРФ — ценные мишени для разработки лекарственных веществ. Уже сейчас доступны препараты для лечения ожоговых и язвенных ран, разрабатываются регенерирующие и противоопухолевые средства [1].

**Figure:**
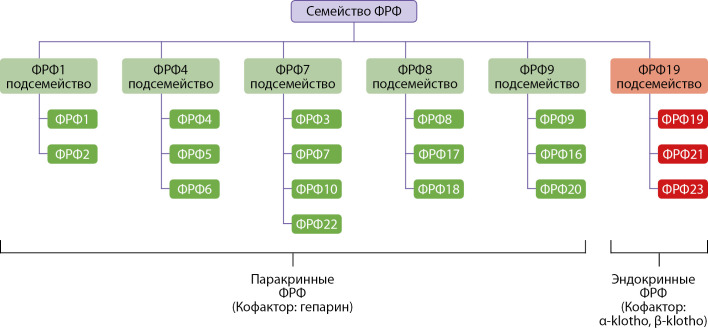
Рисунок 1. Структура семейства ФРФ. Описание. ФРФ млекопитающих классифицируются на шесть подсемейств, при этом эндокринными свойствами обладает лишь одно подсемейство, включающее ФРФ 19, 21, 23. Паракринные ФРФ связываются с рецепторами ФРФР 1–4 и активируют их с помощью кофактора гепарин/гепаринсульфат. Эндокринные ФРФ связываются с рецепторами ФРФР 1–4 и активируют их с помощью кофактора из семейства Клото. Адаптировано Hui Q, Jin Z, Li X, Liu C, Wang X. FGF Family: From Drug Development to Clinical Application. Int J Mol Sci. 2018;19(7):1875. doi: https://doi.org/10.3390/ijms19071875.

## ФРФ 19

У человека ФРФ 19 синтезируется преимущественно в желудочно-кишечном тракте (ЖКТ): в эпителии желчного пузыря, подвздошной кишке, в в печени. Концентрация ФРФ 19 в желчи превышает таковую в системном кровотоке в 20–100 раз. Ген ФРФ 19 (FGF19) локализован в хромосоме 11 (11q13.1), состоит из 216 аминокислот, гомологичен мышиному белку FGF15. Связь с рецепторами разнообразна. Существует высокоспецифичная связь с ФРФ Р4. В присутствии β-клото (β-klotho) ФРФ 19 активирует и другие рецепторы, т.е. ФРФР 1–3. ФРФ 19 преимущественно воздействует на печень, являющуюся органом-мишенью, т.к. в ней экспрессируются высокоспецифичный рецептор ФРФР4 и основной кофактор β-klotho. Внутриклеточная передача сигнала включает в себя активацию mTOR1 — и ERK-зависимых сигнальных путей [12].

ФРФ 19 участвует в распределении питательных веществ, его секреция значительно возрастает через 90–120 минут после приема пищи, а также его называют «гормоном сытости». Экзогенными стимуляторами секреции ФРФ 19 являются жиры и углеводы. Эндогенные регуляторы секреции ФРФ 19 разнообразны, но самым многообещающим с точки зрения возможных лечебных подходов является путь активации секреции ФРФ 19 через фарнезоидный рецептор X (FXR). FXR является одним из рецепторов желчных кислот (ЖК), а при его стимуляции транскрипция гена FGF19 возрастает [3]. На активность ФРФ 19 также могут влиять колебания в экспрессии белка Клото. Еще одним механизмом индукции эффектов ФРФ 19 является изменение печеночной продукции β-klotho. Индукция осуществляется через стимуляцию FXR, а ингибиторами продукции β-klotho являются miRNA-34a, IL-1bв печени и TNF-a в адипоцитах [4]. Наконец, имеет место конкуренция между ФРФ 21 и ФРФ 19 за корецептор Клото.

ФРФ 19 играет важную роль в регуляции количества и состава желчи, воздействуя на FXR и мембранный рецептор ЖК (TGR5). Сами ЖК могут модулировать экспрессию FGF 19, благодаря их связыванию с FXR. Степень индукции экспрессии варьирует у разных ЖК: хенодезоксихолевая, гликодезоксихолевая и холевая кислоты оказывают максимальный эффект, литохолевая и обетихолевая — умеренный, урсодезоксихолевая — слабый. По принципу отрицательной обратной связи сам ФРФ 19 ингибирует секрецию ЖК, подавляя активность CYP7A1 — ключевого фермента классического пути синтеза ЖК. ФРФ 19 влияет и на состав желчи, блокируя CYP7A1, направляет синтез ЖК по альтернативному пути [3]. ФРФ 19 оказывает влияние на объем желчи. У мышей с нефункциональным ФРФ 15 (аналогичен человеческому ФРФ 19) объем желчи в желчном пузыре снижался, а после введения ФРФ 15/19 повышался более, чем в 10 раз [5]. Результаты, полученные у мышей, были подтверждены и в исследованиях на человеке [3].

ФРФ 19 участвует в распределении питательных веществ. Так, на мышах было показано, что экзогенное введение ФРФ 19 усиливает синтез белка и гликогена [6]. Существуют исследования о взаимосвязях между ФРФ 19 и инсулином. В целом, инсулин потенцирует действие ФРФ 19. Инсулин увеличивает уровень ФРФР4 в печени, тем самым потенцируя действие ФРФ 19 на секрецию ЖК [3]. Действие инсулина и ФРФ 19 сходно, но осуществляется через разные внутриклеточные механизмы: инсулин действует преимущественно через систему фосфатинозитол-3-киназа/Akt/Tor/p70S6K, а ФРФ 19 — через сигнальный путь ERK (Ras-ERK, MAPK/ERK) [6]. В постпрандиальном периоде ФРФ 19 подавляет глюконеогенез и цикл трикарбоновых кислот [7]. ФРФ 19 проявляет некоторый антагонизм по отношению к инсулину. Так, он, в отличие от инсулина, при однократном введении не увеличивает количество триглицеридов в печени [6] и даже подавляет экспрессию ферментов липогенеза, увеличивает активность сигнального белка и активатора транскрипции из семейства белков STAT (Signal Transducer And Activator Of Transcription 3 (STAT3)), снижает экспрессию гамма-рецептора, активируемого пролифератором пероксисом, бета-коактиватор 1 (Peroxisome Proliferator-Activated Receptor Gamma, Coactivator 1 Beta (PGC1β)), увеличивает экспрессию ферментов окисления жирных кислот, ингибирует экспрессию ферментов цикла монокарбоновых кислот [3]. У пациентов с сахарным диабетом 2 типа (СД2) регистрируются более низкие показатели циркулирующего ФРФ 19 независимо от массы тела, причем уровни ФРФ 19 и глюкозы натощак находятся в обратной зависимости друг от друга [3]. В долгосрочной перспективе ФРФ 19 способствует снижению накопления липидов в печени.

ФРФ 19 оказывает влияние на энергетический обмен, увеличивая интенсивность расхода энергии, что сопровождается повышением потребления кислорода, улучшением чувствительности к инсулину, повышением утилизации глюкозы и липидов, снижением содержания жировой ткани в организме. Также сообщается, что ФРФ 19 увеличивает выработку адипонектина адипоцитами, тем самым снижая выраженность НАЖБП. Вероятно, ФРФ 19 и адипонектин взаимно регулируют уровень друг друга по реципрокному принципу. У лиц с ожирением наблюдаются более низкие уровни базального ФРФ 19 в крови, коррелирующие с выраженностью висцерального ожирения, причем ассоциации с уровнем глюкозы крови или инсулинорезистентностью не наблюдается [8].

Эффекты ФРФ 19 на центральную нервную систему проявляются в усилении гликолиза, торможении глюконеогенеза, повышении секреции инсулина и подавлении секреции глюкагона, уменьшении потребления пищи, снижении массы тела [7]. Эксперименты с введением ФРФ 19 в различные отделы центральной нервной системы (ЦНС) грызунов выявили новые механизмы центральной регуляции углеводного обмена. В частности, важную роль в ФРФ 19 — опосредованном снижении уровня гликемии играет гипоталамус, экспрессирующий как белок Клото, так и рецепторы ФРФР 1-го (Fibroblast growth factor receptor 1( FGFR1)) и 4-го (Fibroblast growth factor receptor 4 (FGFR4)) типов, и участки заднего мозга, включающие в себя интегративный центр вегетативной нервной системы [3]. Введение ФРФ 19 в латеральный желудочек мозга мыши вызывает столь же выраженное действие на метаболизм, как и инъекции в периферическое кровяное русло, и предполагается, что именно ЦНС играет ключевую роль в реализации системных эффектов ФРФ 19. В частности, расход энергии увеличивается из-за активации симпатической нервной системы. Положительное влияние на углеводный обмен осуществляется, вероятно, еще двумя путями: непосредственным модулированием секреции инсулина и глюкагона и через подавление активности гипоталамо-гипофизарно-надпочечниковой оси со снижением уровня АКТГ и кортизола [9].

ФРФ 19 влияет на мышечную ткань и способен увеличивать количество крупных мышечных волокон [10]. Введение ФРФ 19 молодым и пожилым мышам, вскормленным богатой жирами диетой, помимо известных позитивных эффектов на углеводный и липидный обмены и массу тела, приводило к улучшению силы захвата, препятствовало потере мышечной массы, снижало экспрессию маркеров мышечной атрофии [11].

Имеются данные о влиянии ФРФ 19 на костную ткань [12]. ФРФ 19 способен усиливать остеогенную дифференцировку посредством активации Wnt/β-катенин сигнального пути, ингибировать остеокластогенез через путь остеопротегерин (OPG)/активатор рецептора лиганда NF-κB (RANKL), за счет чего замедляется потеря костной массы при ожирении [12]. При исследовании 73 пациентов старше 60 лет уровень ФРФ 19 был ассоциирован с более высокой минеральной плотностью кости (МПК) [13].

ФРФ 19 участвует в эмбриогенезе: он необходим для развития внутреннего уха, органа зрения, нервной системы, сердца [3].

ФРФ 19 обладает онкогенным действием. Он является мощным стимулятором пролиферации гепатоцитов. Этот эффект становится основой онкогенного влияния ФРФ 19 — при избытке повышается риск развития гепатоцеллюлярной карциномы и опухолей другой локализации, а стимуляция пролиферации опосредована именно высоким количеством ФРФР4 на поверхности печени [3]. С другой стороны, пролиферативный эффект необходим для регенерации печеночной ткани при повреждении печени, в том числе при воздействии канцерогенов. Недостаток ФРФ 19 может привести к повреждению печени вследствие гиперпродукции ЖК и их внутрипеченочного накопления [14].

## ФРФ 21

Фактор роста фибробластов 21 (ФРФ21) — это белок, состоящий из 208 аминокислот и локализующийся на хромосоме 19 (19q13.33). Ген ФРФ21 (FGF21) в организме человека наиболее экспрессирован в печени, жировой ткани, поджелудочной железе, сердечной мышце [15]. ФРФ 21 — это сигнальная молекула, которая связывает печень, ЦНС и жировую ткань, когда необходимо скорректировать расход энергии. ФРФ 21 синтезируется печенью в ответ на пищевые сигналы и с кровотоком достигает ЦНС. Там он оказывает свое основное действие: приводит к смене пищевых приоритетов и, путем центральной бета-адренергической стимуляции, усиливает термогенез в бурой жировой ткани (БуЖТ). Таким образом, ФРФ 21 является важнейшим фактором преодоления окислительного стресса и митохондриальной дисфункции [16].

Секреция ФРФ 21 усиливается при пищевом стрессе (при голодании, ограничении белка, кетогенной или высокоуглеводной диете), физической нагрузке и в иных стрессовых условиях. В спокойном состоянии его уровень практически не определяется. Активация происходит при участии факторов транскрипции Activating Transcription Factor 4 (ATF4) и Nuclear Respiratory Factor (NRF). Кроме того, инициируют секрецию ФРФ 21 углеводы, особенно фруктоза [17].

ФРФ 21 осуществляет свои эффекты через связывание с рецепторами ФРФР1c в присутствии β-klotho. После активации рецептора запускается сигнальный митоген-активируемый протеинкиназный путь (Ras/Raf/MAPK). MAPK индуцирует внеклеточные сигнальные киназы ERK1 и ERK2, которые проникают в ядро и стимулируют транскрипцию генов-мишеней [18]. Помимо этого, ФРФ 21 также активирует 5’АМФ-активируемую протеинкиназу (AMP-activated protein kinase (AMPK) and the histone/protein deacetylase SIRT1 (AMPK-SIRT1)) и сигнальный путь, который индуцирует посттрансляционную модификацию белков [18]. Найдены факторы, которые изменяют взаимодействие ФРФ 21 с рецептором и его эффекты. Так, при ожирении наблюдается резистентность к ФРФ 21. Это обусловлено, во-первых, сниженной экспрессией ФРФР1c в жировой ткани при ожирении. Во-вторых, сниженной экспрессией β-klotho из-за избыточного выброса TNF-α из адипоцитов [19]. Напротив, тиазолидиндионы и глюкагоноподобный пептид-1 (GLP-1) увеличивают экспрессию β-klotho и передачу сигналов ФРФ 21. Локально секреция ФРФ 21 регулируется белком YIPF6 (YIP1 Family Member 6) — мембранным рецептором на секреторных пузырьках эндоплазматического ретикулума [20], который ограничивает секрецию ФРФ 21. В кровотоке ФРФ 21 подвергается протеолитическому расщеплению сывороточными сериновыми протеазами — белком активации фибробластов (FAP) и дипептидилпептидазой IV (DPP-IV) [21].

ЦНС координирует действия ФРФ 21. В ответ на пищевые стимулы печень секретирует ФРФ 21 в кровоток, который достигает ЦНС, в том числе головного мозга, и предоставляет информацию о системном статусе питательных веществ. Сигнал обрабатывается глутаминергическими нейронами вентромедиального гипоталамуса (ВМГ), которые подавляют потребление сахарозы в ответ на повышенную концентрацию глюкозы в плазме. ЦНС координирует дальнейшие действия ФРФ 21: подавление потребления углеводов и алкоголя, увеличение физической активности, усиление сигналов от ЦНС к БуЖТ. Это комплексное действие обеспечивает защиту от увеличения веса. ФРФ 21 контролирует предпочтения в отношении макроэлементов, подавляя аппетит к простым углеводам, а исследования общегеномных ассоциаций (GWAS) идентифицировали однонуклеотидные полиморфизмы (SNP) в гене FGF21, ассоциированные с повышенным предпочтением сладкого вкуса [22]. У людей потребление сахара, особенно фруктозы, является основным стимулом печеночной секреции ФРФ 21. Попадая в ВМГ ЦНС, ФРФ 21 снижает возбудимость глутаминергических нейронов, чувствительных к высокому содержанию глюкозы, таким образом снижается предпочтение сладкого вкуса и уменьшается потребление углеводов [23].

Сахароснижающий эффект ФРФ 21 был открыт в 2005 г. Харитоненковым [25]. Снижение уровня глюкозы в плазме крови на фоне введения ФРФ 21 происходит за счет повышения чувствительности БуЖТ к инсулину. Активация симпатической нервной системы ведет к тому, что белая жировая ткань приобретает некоторые черты бурой жировой ткани, происходит усиление термогенеза и, соответственно, расхода энергии. Помимо активации гипоталамо-гипофизарно-надпочечниковой оси, ФРФ 21 активирует ось «гипоталамус-гипофиз-щитовидная железа» [24]. В жировой ткани (как в бурой, так и в белой) ФРФ 21 секретируется локально и оказывает ауто- и паракринное действие. Помимо стимуляции захвата глюкозы, ФРФ 21 повышает чувствительность жировой ткани к инсулину. По-видимому, ФРФ 21 индуцирует поглощение глюкозы в адипоцитах, в зависимости от действия инсулина. ФРФ 21 индуцирует функцию переносчика глюкозы (Glucose Transporter 1 (GLUT-1)). ФРФ 21 обладает мощным инсулиносенсибилизирующим действием на периферические ткани [21]. Так, у мышей инъекции ФРФ 21 и инсулина существеннее улучшали инсулинозависимое выведение глюкозы из плазмы, чем инъекции одного инсулина [26]. Интересно, что мыши, лишенные жировой ткани, не проявляют этого эффекта [27]. Аналогичным образом удаление β-klotho из адипоцитов полностью устраняло гипогликемическое действие ФРФ 21, а удаление β-klotho из печени — нет [26]. Это говорит о том, что ФРФ 21 проявляет свои гипогликемические свойства главным образом за счет усиления периферической утилизации глюкозы и в меньшей степени влияет на выработку глюкозы печенью. До конца не ясно, центральное или периферическое действие ФРФ 21 становится первопричиной сахароснижающего воздействия. В целом зарегистрированы следующие эффекты: снижение уровня инсулина, активация оси «гипоталамус-гипофиз-надпочечники», изменение циркадианных ритмов поведенческой активности и подавление овуляции путем нарушения передачи сигналов вазопрессин-кисспептин и ингибирования секреции ЛГ [21].

ФРФ 21 стимулирует термогенез. В бурой жировой ткани известны 2 пути. Во-первых, это нисходящий эффект центральной бета-адренергической сигнализации. Во-вторых, это прямое действие на адипоциты, где ФРФ 21 повышает активность рецепторов, активируемых пероксисомными пролифераторами (Peroxisome proliferator-activated receptors, PPARs) [28]. В бурых адипоцитах ФРФ 21, помимо термогенеза, оказывает острое сенсибилизирующее действие к инсулину, индуцируя экспрессию разобщающего митохондриального белка 1 (Mitochondrial brown fat uncoupling protein 1 (UCP-1)) [29]. Белая жировая ткань (в отличие от БуЖТ) не влияет на сахароснижающий эффект ФРФ 21, а подавляет липолиз и, вероятно, стимулирует секрецию адипонектина. Адипонектин уменьшает накопление межклеточных липидов, в первую очередь керамидов у чувствительных к инсулину пациентов. Фармакологические дозы ФРФ 21 индуцируют секрецию адипонектина в белой жировой ткани [30]. Внутрипеченочное накопление керамидов способствует развитию липотоксичности и резистентности к инсулину (ИР) [31]. В целом необходимо дальнейшее изучение взаимосвязи ФРФ 21 и адипонектина в физиологических условиях in vivo.

ФРФ 21 влияет на обмен липидов. Свободные жирные кислоты (СЖК) активируют транскрипцию гена FGF21, воздействуя на сигнальный, ядерный рецептор PPARa, который присутствует в печени. Печень синтезирует ФРФ 21, а далее ФРФ 21 увеличивает экспрессию PPARγ-коактиватора-1α (PGC-1α), ключевого фактора, который способствует окислению СЖК посредством митохондриального биогенеза и усиления функций [7]. PGC-1α направляет СЖК по пути бета-окисления и кетогенеза с образованием ацетоацетата и бета-гидроксибутирата, которые впоследствии используются в качестве источника энергии. ФРФ 21 также усиливает экспрессию митохондриальных генов бета-окисления, то есть Carnitine Palmitoyltransferase 1A (CPT-1α) и 3-Hydroxy-3-Methylglutaryl-CoA Synthase 2 (HMGCS2) [32]. Одновременно подавляется альтернативный путь превращения СЖК в диацилглицерин и триглицериды (ТГ). Снижение концентрации ТГ в сыворотке крови происходит за счет влияния ФРФ 21 на липогенез в печени, подавления липолиза в белой жировой ткани и увеличения активности липопротеинлипазы и катаболизма липопротеинов [21]. Таким образом, ФРФ 21 уменьшает накопление ТГ в печени, предотвращает воспаление и фиброз печени, вызванные избыточным отложением липидов, и защищает другие ткани от метаболического и пищевого стресса (рис. 2, 3). Аналоги ФРФ 21 снижают содержание жира в печени и нормализуют биохимические маркеры цирроза печени у пациентов с ожирением, СД2 и неалкогольным стеатогепатитом (НАСГ) [17].

**Figure:**
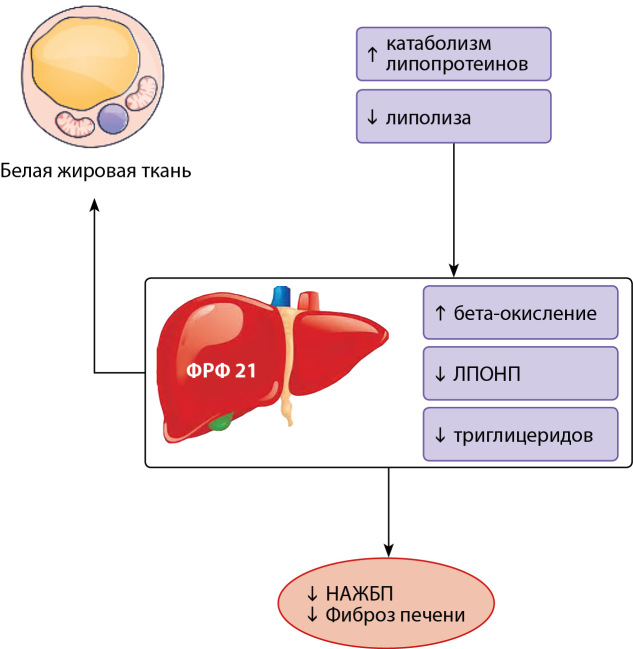
Рисунок 2. Эффекты ФРФ 21. Описание. Белок ФРФ 21 регулирует чувствительность к инсулину и энергетический гомеостаз всего организма. У людей ФРФ 21 вырабатывается главным образом в печени, главный стимул — потребление простых углеводов. С кровотоком ФРФ 21 достигает ЦНС, где снижает возбудимость глюкозозависимых нейронов вентромедиального гипоталамуса (ВМГ), что приводит к подавлению желания сладкого вкуса и снижению потребления сахаров. В бурой жировой ткани ФРФ 21 резко и выраженно повышает чувствительность к инсулину, способствуя утилизации глюкозы, а также способствует выработке тепла во время термогенеза, что объясняет выраженный сахароснижающий эффект. В белой жировой ткани ФРФ 21 повышает чувствительность к инсулину и подавляет липолиз. Адаптировано Szczepańska E, Gietka-Czernel M. FGF21: A Novel Regulator of Glucose and Lipid Metabolism and Whole-Body Energy Balance. Horm Metab Res. 2022;54(4):203-211. doi: https://doi.org/10.1055/a-1778-4159.

**Figure:**
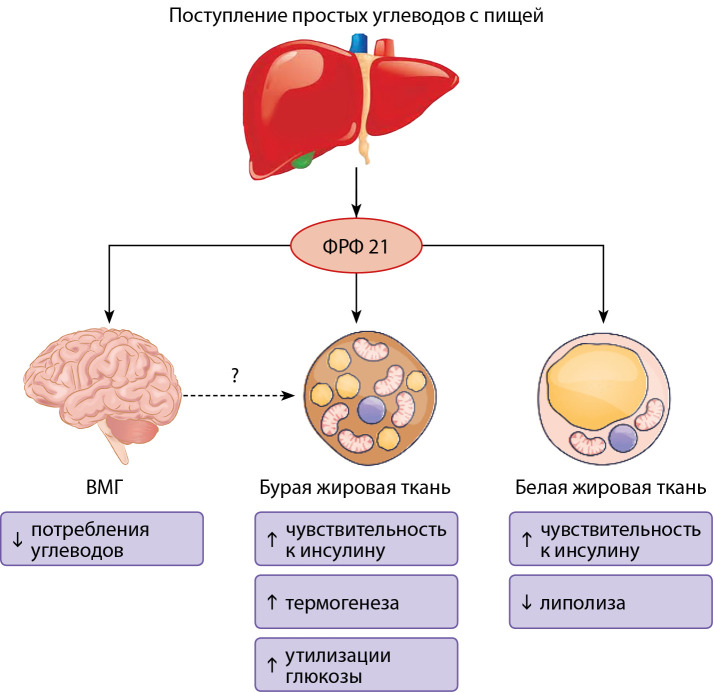
Рисунок 3. Эффекты ФРФ 21. Описание. Белок ФРФ 21 препятствует жировой дистрофии печени и фиброзу. ФРФ 21 действует локально в печени, стимулируя β-окисление свободных жирных кислот и подавляя образование триглицеридов и выработку ЛПОНП. ФРФ 21 уменьшает поступление липидов в печень, индуцируя катаболизм периферических липопротеидов и подавляя липолиз жировой ткани. В итоге ФРФ 21 снижает содержание внутрипеченочных и сывороточных триглицеридов. Введение ФРФ 21 приводит к регрессу НАЖБП и фиброза печени. ЛПОНП — липопротеины очень низкой плотности; НАЖБП — неалкогольная жировая болезнь печени. Адаптировано Szczepańska E, Gietka-Czernel M. FGF21: A Novel Regulator of Glucose and Lipid Metabolism and Whole-Body Energy Balance. Horm Metab Res. 2022;54(4):203-211. doi: https://doi.org/10.1055/a-1778-4159.

ФРФ 21 оказывает множество полезных метаболических эффектов, однако его повышение наблюдается при патологических состояниях, в частности при инсулинорезистентности, ожирении, СД2 и неалкогольной жировой болезни печени (НАЖБП) и стеатозе печени. Вероятно, это является компенсаторной реакцией или нечувствительностью тканей к сигналам ФРФ 21.

Данные об эффектах ФРФ 21 в костной ткани крайне противоречивы. Есть свидетельства, что в зрелых остеокластах экспрессируется как ФРФР, так и β-klotho. На животных моделях показано, что влияние ФРФ 21 реализуется через активацию PPARγ, что ведет к усилению костной резорбции и снижению костеобразования вследствие ингибирования остеобластогенеза. В исследованиях на мышах было показано, что ФРФ 21 может воздействовать на остеоциты, приводя к более высокому соотношению RANKL/OPG по сравнению с мышами с нокаутированным ФРФ 21 [33].

Мышцы демонстрируют очень ограниченную экспрессию ФРФ 21 в физиологических условиях. Однако его синтез резко возрастал при митохондриальных миопатиях вследствие чрезмерного окислительного стресса [34].

Благоприятные метаболические эффекты ФРФ 21 сделали его привлекательной лекарственной мишенью для лечения ожирения, СД2, дислипидемии, НАЖБП. Нативный ФРФ 21 не подходит для терапевтического применения из-за фармакодинамических свойств: короткий период полувыведения 0,5–1 час, расщепление сывороточными протеазами, склонность к выпадению в нерастворимый осадок. Были синтезированы аналоги, устойчивые к агрегации или протеолитическому расщеплению. Кроме того, разработали агонисты рецептора ФРФ 21, например, моноклональные антитела, связывающиеся с комплексом ФРФР1-β-klotho, или искусственные белки, связывающие специфические антигены, активирующие ФРФР1-β-klotho [16]. Аналоги ФРФ 21 планировалось использовать как препараты для лечения СД2, однако они не вызвали значительного гипогликемического эффекта. При этом обнаружилось их выраженное гиполипидемическое действие, способность повышать уровень адипонектина и снижать вес. Миметики ФРФ 21 перспективны для лечения НАЖБП. В клиническом исследовании аналога ФРФ 21 пегбельфермин IIa фазы у пациентов с неалкогольным стеатогепатитом (НАСГ) наблюдалось значительное снижение уровня N-концевого пропептида коллагена III типа (PRO-C3) в сыворотке крови — маркера фиброза печени, но не было отмечено снижения уровня гликированного гемоглобина (HbA1c) [35]. В другом исследовании пегбельфермина у пациентов с НАСГ выявили снижение содержания жира в печени, улучшение биохимических показателей фиброза печени (Про-С3, АЛТ, АСТ), повышение уровня адипонектина в сыворотке крови [36]. Существуют опасения по поводу побочных эффектов аналогов ФРФ 21 (AKR-001 и NGM-313) при длительном применении, поэтому сейчас ведутся исследования их безопасности и эффективности. В частности, сообщалось, что у мышей лечебные дозы ФРФ 21 приводили к потере костной массы [37], у человека наблюдалось увеличение маркеров костной резорбции, а также появление ФРФ 21-антител у 50% пациентов, получивших пегбелфермин [35]. С другой стороны, аналоги ФРФ 21 хорошо переносятся, и большинство побочных эффектов возникают со стороны ЖКТ.

Таким образом, попытки применения аналогов ФРФ 21 показали множество положительных метаболических эффектов, что является привлекательной и перспективной стратегией лечения заболеваний, связанных с ожирением, гиперхолистеринемией и гипертриглицеридемией, отложением липидов в печени, повышением уровня глюкозы крови. Однако необходимы клинические испытания с оценкой долгосрочных эффектов аналогов ФРФ 21.

## ФРФ 23

Белок ФРФ 23 был открыт в 2000 г. японским исследователем Yamashita T Была доказана его роль в патогенезе аутосомно-доминантного рахита, oпухоль-индуцированной остеомаляции и при почечной недостаточности (ХБП) [38]. Ген ФРФ 23 (FGF23) располагается на 12-й хромосоме (12p13) и состоит из 3 экзонов. Клетки костной ткани (преимущественно остеоциты) синтезируют белок, состоящий из 251 аминокислоты (32 kD). Однако небольшое количество ФРФ 23 синтезируют и другие ткани, такие как селезенка, тимус, почки, печень, сердце, мозг, тонкий кишечник [1]. Регуляция концентрации ФРФ 23 осуществляется на множестве биологических уровней (в процессе транскрипции, посттрансляционно, в системном кровотоке), что свидетельствует о его важности для организма. На процесс транскрипции ФРФ 23 оказывают влияние белки: Матриксный белок дентина-1 (Dentin Matrix Acidic Phosphoprotein 1 (DMP-1)), Регулятор переноса неорганического пирофосфата (ANKH Inorganic Pyrophosphate Transport Regulator (ANKH)), фосфат-регулирующей эндопептидазы (Phosphate Regulating Endopeptidase X-Linked (PHEX)), экто-нуклеотид-пирофосфатаза/фосфодиэстераза (Ectonucleotide Pyrophosphatase/Phosphodiesterase 1 (ENPP1)), а мутации в их генах приводят к врожденным заболеваниям и рахиту. Посттрансляционные модификации в норме регулируют концентрацию ФРФ 23 в крови. Это осуществляется частичной инактивацией ФРФ 23 внуриклеточно, путем протеолиза ФРФ 23 белком «фурин-подобная киназа», ассоциированным с секреторным путем Гольджи (FURIN-like proteasa Golgi Associated Secretory Pathway Kinase (FAM20C)), а восприимчивость ФРФ23 к протеолизу регулируется о-гликозилированием белком «Полипептидная N-ацетилгалактозаминилтрансфераза 3» (Polypeptide N-Acetylgalactosaminyltransferase 3) GalNAc-N3 (GALNT3)) [38].

В крови ФРФ 23 может определяться в нескольких формах. Это биоактивный «интактный ФРФ 23» (N+C -пептиды) и небиоактивные, короткие С-пептид и N-пептид. Современные лабораторные наборы в большинстве случаев определяют интактный ФРФ 23. Однако есть тесты, которые оценивают С-пептид. Исследуются соотношения форм ФРФ 23 в крови, что может помочь в диагностике ФРФ 23-обусловленных нарушений. В системном кровотоке концентрации ФРФ 23 регулируются предположительно ингибиторами активатора плазминогена (plasminogen activator inhibitor–1 (PAI-1)) [38].

Активно изучается влияние на концентрацию ФРФ 23 системных факторов: 1,25 дигидроксихолекальциферола (1,25(OH)2D, кальцитриол), паратиреоидного гормона (ПТГ), фосфора, кальция, растворимого в сыворотке белка Клото (serum soluble Klotho, (sKlotho)), факторов воспаления, дефицита железа и эритропоэтина. 1,25(OH)2D, ПТГ, фосфор, кальций, повышают концентрацию ФРФ 23. Так, ПТГ стимулирует синтез ФРФ 23, способствует превращению 25-гидроксивитамина D3 в его активный метаболит 1a,25(OH)2D3, который, в свою очередь, увеличивает всасывание фосфата в кишечнике, что также стимулирует синтез ФРФ 23 [39]. Дефицит железа может параллельно увеличить как транскрипцию ФРФ 23, так и его расщепление [38]. Системное воспаление характеризуется низким содержанием железа в сыворотке крови, несмотря на нормальные или повышенные запасы железа в организме, что приводит к аналогичному влиянию на ФРФ 23 (рис. 4).

**Figure:**
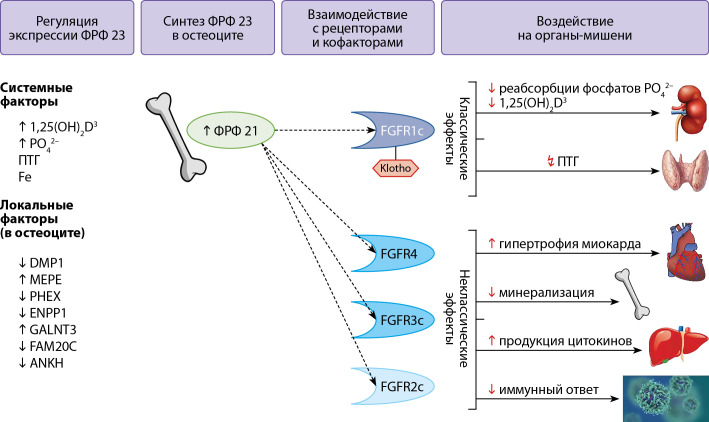
Рисунок 4. Эффекты ФРФ 23. Описание. Белок ФРФ 23 (251 амк) синтезируется в остеоцитах. Процесс контролируется множественными факторами, как системными (концентрациями фосфатов, ПТГ, 1,25(OH)2D3), так и локальными белками в остеоцитах. Процесс транскрипции ингибируют белки DMP-1, ANKH, PHEX, ENPP1. Процессы посттрансляционной модификации регулирует белок GalNAc-T3 (продукт гена GALNT3), препятствуя инактивации ФРФ 23, а белок Fam20C (FAM20C) наоборот расщепляет ФРФ 23, делая его неактивным. В системном кровотоке ФРФ 23 взаимодействует с рецепторами и кофакторами. Самые изученные, фосфатурические эффекты ФРФ 23 названы классическими (или каноническими) и осуществляются при взаимодействии с рецептором FGFR1c и корецептором α-Клото. Снижая реабсорбцию фосфатов, ингибируя синтез кальцитриола, регулируется баланс фосфора в организме. Классические эффекты ФРФ 23 осуществляются, в частности, через сигнальный путь FRS2/RAS/RAF/MEK/ERK1/2. Изучаются новые эффекты ФРФ 23, которые осуществляются через взаимодействие с другими рецепторами (FGFR2/3/4) на миокард, печень, иммунные клетки, костный мозг. Неклассические эффекты ФРФ 23 осуществляются, в частности, через сигнальный путь FGFR3/FGFR4/calcineurin/NFAT. ФРФ23 — фактор роста фибробластов 23; GalNAc-T3 — полипептид N-ацетилгалактозаминилтрансфераза 3; 1,25(OH)2D3 — 1,25 дигидрокси Д3 витамин или кальцитриол; PO42 — неорганический фосфат, DMP-1 — белок дентинного матрикса-1; MEPE — матриксный внеклеточный фосфогликопротеин; PHEX — фосфатрегулирующая нейтральная эндопептидаза на хромосоме X; ANKH — гомолог белка прогрессирующего анкилоза; ENPP1 — эктонуклеотидпирофосфатаза/фосфодиэстераза 1; Fam20c — серинкиназа, локализованная в аппарате Гольджи; Fe — сывороточное железо; ПТГ — паратгормон.

Классическим, или каноническим эффектом ФРФ 23 является контроль уровня фосфора. ФРФ 23 регулирует баланс фосфатов, удаляя избыток фосфора с мочой, снижая активность натрий-зависимых фосфатных транспортеров ((Sodium-dependent phosphate transport protein 2A (NaPi2a), Sodium-dependent phosphate transport protein 2С (NaPi2с)), тем самым повышая экскрецию фосфора. Кроме того, ФРФ 23 ингибирует синтез 1,25(OH)2D в почках, снижая активность D-1α-гидроксилазы (CYP27B1), и усиливает его распад через активацию 24α-идроксилазы (CYP24A1). Фосфатурические эффекты ФРФ 23 осуществляются при взаимодействии с рецептором ФРФР1, подтип С (FGFR1C), и требуют обязательного наличия корецептора, трансмембранного белка альфа-Клото (α-Klotho) [38]. Органами-мишенями являются почки и паращитовидные железы. В паращитовидных железах ФРФ 23 ингибирует секрецию ПТГ, однако этот эффект зачастую преодолевается ФРФ 23-опосредованной супрессией кальция, что влечет значительное повышение продукции ПТГ и хронически сниженный уровень фосфора, который также стимулирует кальций-чувствительные рецепторы.

С течением времени были изучены новые, названные неклассическими, или неканоническими эффекты ФРФ 23. Эти эффекты ФРФ 23 регулируют воспаление в гепатоцитах [40], индуцируют гипертрофию миокарда [41], ингибируют нейтрофиллез [42]. Также было отмечено, что концентрации ФРФ 23 в плазме возрастали при наличии хронических заболеваний. В частности, при ХБП высокие концентрации ФРФ 23 в плазме наблюдались прежде, чем развивались гиперпаратиреоз или гиперфосфатемия [43]. А также было доказано, что концентрации ФРФ 23 коррелируют с прогрессированием ХБП. При других хронических заболеваниях было показано, что ФРФ 23 ассоциирован с атеросклерозом сонных артерий, прогрессированием фиброза при сердечной недостаточности [44]. Дислипидемия связана с более высокими уровнями ФРФ 23. Воспаление также повышает концентрацию ФРФ 23. Следовательно, ФРФ 23 изучается и является потенциальным биомаркером, коррелирующим с прогрессированием и исходом некоторых серьезных хронических заболеваний.

Помимо физиологической секреции, ФРФ 23 описан как гормон паранеопластической секреции некоторыми опухолями, что приводит к клинической картине фосфопенической остеомаляции. Опухоль-индуцированная остеомаляция — это редкий паранеопластический синдром, развивающийся в результате гиперэкспрессии ФРФ 23 и сопровождающийся избыточными потерями фосфора с мочой, снижением концентраций 1,25(OH)2D3. В результате пациент страдает от гипофосфатемии, остеомаляции, деминерализации костей [38, 45]. Остеомаляция, индуцированная опухолью, наиболее часто возникает из-за приобретенной доброкачественной мезенхимальной опухоли, способной секретировать ФРФ 23 [46–48]. В редких случаях может быть связана со злокачественными новообразованиями, такими как рак предстательной железы, рак легкого, рак яичников, аденокарцинома, анапластический рак щитовидной железы, B-клеточная лимфома Ходжкина, рак груди и внутричерепные опухоли [49–52]. Все они являются немезенхимальными опухолями, но претерпевают эпителиально-мезенхимальный переход в процессе метастазирования. Симптомы, характерные для фосфопенической остеомаляции опухолевого генеза, у пациентов с онкологическими заболеваниями могут быть расценены как прогрессирование онкологического процесса. Поэтому мониторинг фосфора и кальция оптимально проводить у всех пациентов с костными метастазами андроген-резистентных форм рака предстательной железы для своевременного выявления и коррекции гипофосфатемии. Было показано, что ФРФ 23 действует как аутокринный фактор в клетках рака предстательной железы, стимулируя инвазию опухоли и клеточную пролиферацию, а выработка ФРФ 23 аутокринно стимулируется через рецептор ФРФР1. Кроме того, клетки рака предстательной железы могут стимулировать экспрессию ФРФ 23 в остеоцитах, хотя уровень ФРФ 23 в плазме крови не всегда изменяется [50].

Опухолевые очаги в костях могут быть подвержены влиянию ФРФ 23. В 2019 г. Mansinho et al. показали, что высокие концентрации ФРФ 23 ассоциированы с низкой выживаемостью пациентов с костными метастазами, и сделали вывод, что передача сигналов ФРФ 23 может непосредственно способствовать прогрессированию заболевания [53]. Соответственно, лечение анти-ФРФ 23 антителом может оказывать благоприятный эффект и должно быть изучено. В 2020 г. Weidner et al. показали, что пациенты с миелодиспластическим синдромом и нарушением кроветворения в костном мозге имеют более высокую концентрацию ФРФ 23 [54]. Suvannasankha et al. в 2015 г. показали, что при множественной миеломе и поражении костей клетки демонстрируют Клото-зависимую передачу сигналов ФРФ 23, а уровни интактного ФРФ 23 в плазме повышены [55].

При раке эндометрия и гинекологических опухолях чаще не наблюдается изменений концентрации ФРФ 23 в плазме [56]. Рак молочной железы может быть связан с онкогенной остеомаляцией и повышенным уровнем ФРФ 23. Экспрессия мРНК ФРФ 23 высока в клетках рака молочной железы, и ФРФ 23, продуцируемый опухолевыми клетками, способствует прогрессированию метастатического поражения и онкогенезу. При других опухолевых образованиях концентрация ФРФ 23 в крови повышается, однако биологический смысл этого не ясен, а дальнейшие исследования этой области остаются актуальны.

## ЗАКЛЮЧЕНИЕ

За последнее десятилетие были достигнуты большие успехи в изучении эндокринных свойств ФРФ 19, 21 и 23. Это открывает новые перспективы для медицины. Современные знания частично проясняют сложную биологическую роль ФРФ 23 в регуляции обмена фосфора, значение ФРФ 21 в обмене липидов, углеводов и термогенеза, а также роль ФРФ 19 в постпрандиальном метаболизме печени и желчных кислот. Знания о свойствах этих неклассических гормонов легли в основу разработок новых лекарственных препаратов, используемых как для стимуляции эффектов (ФРФ 21, 19), так и подавления влияний ( ФРФ 23). Дальнейшие исследования в данной области представляют значительный потенциал для разработки терапевтических стратегий, направленных как на лечение широко распространенных состояний, таких как ожирение, жировой гепатоз и метаболический синдром, так и на более редкие патологии, например, фосфопеническую остеомаляцию. Ожидается, что такие подходы смогут существенно улучшить как качество, так и продолжительность жизни пациентов.

## Дополнительная информация

Источники финансирования. Государственное задание №124020700097-8.

Конфликт интересов. Авторы декларируют отсутствие явных и потенциальных конфликтов интересов, связанных с публикацией настоящей статьи.

Участие авторов. Гронская С.А. — написание текста статьи, идея обзора, сбор данных и анализ; Русяева Н.В. — написание текста статьи, сбор данных и анализ; Белая Ж.Е. — написание текста статьи, идея обзора, сбор данных и анализ, внесение правок; Мельниченко Г.А. — написание текста статьи, идея обзора, сбор данных и анализ, внесение правок.

Все авторы одобрили финальную версию статьи перед публикацией, выразили согласие нести ответственность за все аспекты работы, подразумевающую надлежащее изучение и решение вопросов, связанных с точностью или добросовестностью любой части работы.
